# Safewards Impact in Inpatient Mental Health Units in Victoria, Australia: Staff Perspectives

**DOI:** 10.3389/fpsyt.2019.00462

**Published:** 2019-07-10

**Authors:** Justine Fletcher, Bridget Hamilton, Stuart A. Kinner, Lisa Brophy

**Affiliations:** ^1^Centre for Mental Health, Melbourne School of Population and Global Health, The University of Melbourne, Melbourne, VIC, Australia; ^2^Centre for Psychiatric Nursing, School of Health Sciences, The University of Melbourne, Melbourne, VIC, Australia; ^3^Melbourne School of Population and Global Health, The University of Melbourne, Melbourne, VIC, Australia; ^4^Centre for Adolescent Health, Murdoch Children’s Research Institute, Heidelberg, VIC, Australia; ^5^Mater Research Institute-UQ, University of Queensland, Brisbane, QLD, Australia; ^6^Griffith Criminology Institute, Griffith University, Mt Gravatt, QLD, Australia; ^7^School of Allied Health, Human Services and Sport, La Trobe University, Bundoora, VIC, Australia; ^8^Mind Australia Limited, Heidelberg, VIC, Australia

**Keywords:** mental health service, safewards, inpatient psychiatry, restrictive practices, recovery oriented care

## Abstract

**Introduction:** Mental health professionals working in acute inpatient mental health wards are involved in a complex interplay between an espoused commitment by government and organizational policy to be recovery-oriented and a persistent culture of risk management and tolerance of restrictive practices. This tension is overlain on their own professional drive to deliver person-centered care and the challenging environment of inpatient wards. Safewards is designed to reduce conflict and containment through the implementation of 10 interventions that serve to improve the relationship between staff and consumers. The aim of the current study was to understand the impact of Safewards from the perspectives of the staff.

**Methods:** One hundred and three staff from 14 inpatient mental health wards completed a survey 12 months after the implementation of Safewards. Staff represented four service settings: adolescent, adult, and aged acute and secure extended care units.

**Results:** Quantitative results from the survey indicate that staff believed there to be a reduction in physical and verbal aggression since the introduction of Safewards. Staff were more positive about being part of the ward and felt safer and more connected with consumers. Qualitative data highlight four key themes regarding the model and interventions: *structured and relevant; conflict prevention and reducing restrictive practices; ward culture change; and promotes recovery principles.*

**Discussion:** This study found that from the perspective of staff, Safewards contributes to a reduction in conflict events and is an acceptable practice change intervention. Staff perspectives concur with those of consumers regarding an equalizing of staff consumer relationships and the promotion of more recovery-oriented care in acute inpatient mental health services.

## Introduction

In Australia and internationally, there has been a movement by consumers and carers, supported in national policy, toward the provision of recovery-oriented care (1, 2). The core of recovery orientation is that consumers, with or without symptoms of mental illness, are central in setting their own priorities for care and receive the necessary support to live a meaningful life of their choosing (3–5).

The current National Mental Health Policy emphasizes reducing use of restrictive practices in inpatient mental health services (6). Research has found that there is no evidence that seclusion is therapeutic (7). One qualitative study found diverse views among staff, some believing seclusion was part of treatment and others believing it was a punishment (8). Emerging evidence, particularly in the qualitative literature, highlights findings from consumers and staff that the use of restrictive practices, including seclusion, can be experienced as retraumatizing for consumers and for those who witness these practices (9–11). The use of restrictive practices can lead to consumers feeling unsafe and may interfere with ongoing personal recovery and engagement with services (11, 12).

Inpatient mental health services are complex environments for people experiencing the most acute symptoms of mental illness. People are often involuntarily admitted for short periods of time, and it has been asserted by some that the focus is on stabilization of a pharmaceutical regime. It has been suggested that staff in inpatient units tend to rely on medication as the primary treatment under a medical model of care (13–15). Organizational safety and risk management provide fundamental guidance to practice. Slemon et al. (16) have argued that the risk management culture that drives care in inpatient mental health settings results in a perpetuation of stigma that people with a mental illness are aggressive. Therefore, staff are responsible for maintaining the safety of everyone in the ward, legitimizing the use of restrictive practices to maintain control and safety (16, 17). Support for this approach is potentially located in findings that mental health professionals are at higher risk of being exposed to physical aggression than many other health care professionals (18). Mental health nurses are often fearful about being injured at work and may as a group feel that the use of restrictive practices (such as seclusion) is necessary (8). Staff also report cognitive dissonance (19) with feelings of guilt associated with forcing consumers to take medication and using restrictive practices but a sense of being trapped in these ways of working (8). Despite this tension, nurses are motivated to engage more therapeutically with patients, yet aspects of the institutional flow, such as short stays and excessive paperwork, discourage engagement. Even so, research has found that nurses who spend more time directly caring for patients experience greater job satisfaction (20).

It has been argued that the challenges inherent in caring for consumers in services that prioritize medication adherence and risk management have resulted in nurses lacking time and autonomy to engage in therapeutically meaningful interactions with consumers, causing frustration for both consumers and nurses (21, 22). The development of a therapeutic relationship is viewed by many as the single most important factor in a positive inpatient admission (12, 23, 24). Despite this, research in one Australian state using a work sampling methodology has found that only 32% of nurse time was spent in direct care (25). A slightly higher proportion of time (42.7%) in direct care was found by Whittington and McLaughlin (26) in an observational UK study; however, the specific measure regarding time spent in potentially therapeutic interactions was observed to be 6.75%. Goulter et al. (25) went on to conclude that the lack of time spent in direct care falls short of the expectations of consumers, and emerging evidence highlights that positive engagement is related to higher levels of consumer satisfaction (27). Furthermore, a review of literature related to the measurement of therapeutic relationships indicates that better quality therapeutic relationships may be achieved by nurses having increased time to spend with consumers, but that research regarding this is lacking (28). To improve this situation, Goulter et al. (25) suggest the need for “a comprehensive model of practice that draws on the best available evidence of what activities constitute best nursing practice in mental health settings” (p. 455). The Safewards model and interventions may provide one avenue of addressing the need expressed by Goulter et al. (25).

Safewards was developed after a series of comprehensive literature reviews and empirical research (29). Safewards offers a multifaceted approach to reducing conflict and the use of containing or restrictive practices by helping to shift the focus of staff back to direct care and building therapeutic relationships (30). Safewards is a theoretical model with 10 associated interventions designed to improve the safety of everyone in inpatient wards by reducing conflict (physical, verbal aggression, absconding) and containment (forced medication, seclusion, and restraint) events. For a full description of the model, see Bowers (30). Informed by extensive literature reviews and empirical research, the Safewards model proposes that six originating domains (the patient community, patient characteristics, regulatory framework, staff team, physical environment, and outside hospital) potentially contribute to flashpoints (e.g., a situation signaling and preceding a conflict event, such as physical aggression), which may then lead to conflict and containment (29). Staff have the potential to moderate each of these components of the model through their interactions with consumers. The interventions are described in **Table 1**.

**Table 1:** Safewards Interventions.

Intervention	Description	Purpose
**Mutual Help Meeting (1)**	Patients offer and receive mutual help and support through a daily, shared meeting.	Strengthens patient community, opportunity to give and receive help
**Know Each Other (1)**	Patients and staff share some personal interests and ideas with each other, displayed in unit common areas.	Builds rapport, connection, and sense of common humanity
**Clear Mutual Expectations (1)**	Patients and staff work together to create mutually agreed aspirations that apply to both groups equally.	Counters some power imbalances, creates a stronger sense of shared community
**Calm Down Methods (1)**	Staff support patients to draw on their strengths and use/learn coping skills before the use of *pro re nata* medication or containment.	Strengthen patient confidence and skills to cope with distress
**Discharge Messages (1)**	Before discharge, patients leave messages of hope for other patients on a display in the unit.	Strengthens patient community, generates hope
**Soft Words (2)**	Staff take great care with their tone and use of collaborative language. Staff reduce the limits faced by patients, create flexible options, and use respect if limit setting is unavoidable.	Reduces a common flashpoint, builds respect, choice, and dignity
**Talk Down (2)**	De-escalation process focuses on clarifying issues and finding solutions together. Staff maintain self-control, respect, and empathy.	Increases respect, collaboration, and mutually positive outcomes
**Positive Words (2)**	Staff say something positive in handover about each patient. Staff use psychological explanations to describe challenging actions.	Increases positive appreciation and helpful information for colleagues to work with patients
**Bad News Mitigation (2)**	Staff understand, proactively plan for. and mitigate the effects of bad news received by patients.	Reduces impact of common flashpoints, offers extra support
**Reassurance (2)**	Staff touch base with every patient after every conflict on the unit and debrief as required.	Reduces a common flashpoint, increases patients’ sense of safety and security

(1) Interventions directly involving consumers.

(2) Interventions requiring active practice change of clinicians

Adapted from the DHHS Safewards flier overview and original material developed by Professor Len Bowers, UK (31).

In a pragmatic cluster randomized controlled trial of Safewards in the United Kingdom, Bowers et al. (32) observed a significant decrease in conflict and containment events in the Safewards condition compared with the control condition where a staff physical health improvement package was offered. However, variable success regarding implementation of Safewards has been reported in recent papers. Problems with implementation have included low adherence (fidelity) and lack of staff acceptance of the model (33, 34). This contrasts with high fidelity to the model in some settings, resulting in reduction in conflict events (35) and reduction in the use of forced sedation (36). Hence, high fidelity to the model is important to its outcomes. To date, the perceptions of staff from wards that have successfully implemented Safewards and therefore contributed to fidelity to the model have not been reported.

Based on the promising randomized controlled trial results from the United Kingdom, in 2014, the Victorian Department of Health in Australia funded seven self-selected health services to implement Safewards across 18 wards in urban and regional Victoria. Our team was commissioned to undertake an independent evaluation across the seven services. We used a pragmatic real-world evaluation design to evaluate training outcomes, impact of Safewards from consumer and staff perspectives, and short-term and long-term outcomes related to implementation fidelity and seclusion rates. The results from adult and youth acute wards suggest a significant reduction in seclusion rates, from 14.1 seclusions per 1,000 occupied bed days pre to 10.1 seclusions per 1,000 occupied bed days at 12 months’ follow-up, representing a 36% reduction (37). At 12-month follow-up, on average, 9 of the 10 Safewards interventions were being implemented (37). Consumer feedback from Victoria highlights that consumers believed that there was a reduction in physical and verbal aggression after implementation of Safewards. Overall, consumers felt safer and reported increased connection with staff and each other, leading to an experience of care that was more in line with a recovery orientation (38). In this paper, we aim to report on staff perspectives that formed part of the overall evaluation findings and compare and contrast with previously reported findings regarding consumer perspectives (38).

## Method

### Design

A cross-sectional postintervention survey design was used to study staff perspectives. Staff were surveyed between December 2015 and April 2016, 9–12 months after Safewards was first implemented, at which time, on average, 9 of the 10 Safewards interventions were implemented.

### Setting

This study is based on inpatient mental health wards in both metropolitan and regional Victoria, Australia. It reports data from six of the seven health services that opted to implement Safewards. The inpatient services were adult, adolescent/youth, and aged acute wards and secure extended care units.

### Participants

Current staff on 14 wards from six of the seven health services that implemented Safewards were invited to take part in the staff survey. One service decided not to take part in the survey of staff.

### Measures

The purpose-designed survey included demographic characteristics and both quantitative and qualitative questions regarding the acceptability, applicability, and impact of the Safewards model and 10 interventions. The survey was developed by the research team in reference to the overarching research questions with further input from the commissioning agency. All members of the research team were trained mental health clinicians who had experience working in inpatient settings alongside their research expertise. The face validity of the items was agreed to by all parties.

Five quantitative questions covered: 1) how suitable staff thought Safewards was using a Likert scale, where 1 = poor, 2 = fair, 3 = good, 4 = very good, 5 = excellent; 2) how frequently interventions were used; 3) would they be sustained over the next 12 months using a Likert scale, where 1 = highly unlikely, 2 = not likely, 3 = possible, 4 = probable, 5 = highly probable; 4) the impact of Safewards on four conflict events (property damage, absconding, physical conflict, and verbal conflict) that were agreed upon by the researchers and the Government team piloting Safewards as the most relevant in the Victorian context at the time; and 5) the impact of Safewards on the atmosphere of the ward. Participants responded to these questions on a 5-point Likert scale. The Likert scale anchor points for questions two, four, and five were 1 = never, 2 = rarely, 3 = sometimes, 4 = usually, 5 = always.

### Procedures

The plain language statement and consent form made clear that participation was voluntary, and that staff could withdraw at any time. The survey was hosted on SurveyMonkey; staff were sent a link from the local Safewards lead via e-mail. Ethics approval was obtained via the Victorian Human Research Ethics Multi-site process (ID 15225L) for each of the involved services.

### Data Analysis

Quantitative data were analyzed descriptively using SPSS version 22. Weighted averages for the Likert scales were calculated using the number of people who selected a given response and the weighting of that response. Staff who rated the Safewards model or one of the interventions as “poor” or “excellent” were given the opportunity to provide a detailed comment. Qualitative data were analyzed by two of the researchers (JF and BH) using a thematic approach guided by the approach outlined by Braun and Clarke (39). We elected to use an inductive process to uncover emerging themes. The steps we took were 1) to become familiar with the data, qualitative comments were read and counted to gain an understanding of the spread of feedback from participants; 2) initial codes were generated about the data, particularly assessing the spread of positive, negative, and neutral comments to provide a sense of the overall perspective of participants about Safewards; 3) comments of 3 or more words (i.e., those with some meaning to be elucidated) were categorized according to emerging themes; 4) we reviewed and where necessary reorganized the data according to the themes; 5) we discussed the names and definitions of each theme to ensure that they captured the essence of the data; and 6) the analysis was written up and examined to ensure accurate representation of the data according to the themes.

## Results

One hundred three staff responded to the survey representing each of the 14 wards. The majority were English-speaking women with a mean age of 43 years (range, 21–61, SD 10.28). Each service type was represented, with secure extended care unit (SECU) being slightly overrepresented and adolescent/youth wards being slightly underrepresented. Fifty-five percent of staff were registered or enrolled nurses, and almost 20% reported being from another professional group, including occupational therapists, social workers, and medical staff (**Table 2**).

**Table 2:** Participant characteristics.

	Frequency	%
**Gender** ***n*** ** = 76**		
Male	22	28.9
Female	52	68.4
Other	2	2.6
**Language** ***n*** ** = 74**		
English	70	94.6
Other	4	5.4
**Service Type** ***n*** ** = 76**		
Adult	42	55.3
Adolescent/youth	4	5.3
Aged	13	17.1
SECU	17	22.4
**Professional Role** ***n*** ** = 72**		
Clinical nurse educator	3	4.2
Nurse unit manager	5	6.9
Associate nurse unit manager	7	9.7
Clinical nurse specialist	3	4.2
Registered nurse	25	34.7
Enrolled nurse	14	19.4
Consumer consultant	1	1.4
Peer worker	0	0.0
Other	14	19.4

SECU, secure extended care unit.

**Table 3** displays the weighted average response according to the suitability of Safewards, the frequency of use in the ward, and the likelihood of the intervention remaining in place over the next 12 months. On average, staff rated the suitability of the Safewards model and interventions as good to very good. Variation among staff may indicate differences in service settings; however, there was not enough data in each group to test this statistically. Staff reported that each of the Safewards interventions was used in their ward on average sometimes to usually. Staff held a positive view that it was probable to highly probable that Safewards would still be in place in their ward in 12 months’ time.

**Table 3:** Suitability, use, and sustainability of Safewards model and interventions.

Model/Intervention	Suitability		Frequency of use in the unit		Sustainability over 12 months	
	*n*	Weighted average	*n*	Weighted average	*n*	Weighted average
Safewards Model	90	3.9			77	4.2
Clear Mutual Expectations	90	3.5	75	3.8	76	4.2
Soft Words	90	3.6	77	4.2	77	4.3
Talk Down	90	4.0	77	4.2	77	4.4
Positive Words	90	3.8	77	4.2	77	4.3
Bad News Mitigation	90	3.5	76	3.6	77	4.2
Know Each Other	90	3.7	78	3.9	76	4.1
Mutual Help Meeting	90	3.5	76	3.8	77	4.3
Calm Down Methods	90	4.0	77	4.3	77	4.4
Reassurance	90	3.8	77	4.3	77	4.3
Discharge Messages	90	3.7	77	3.6	76	4.2

**Figure 1:**
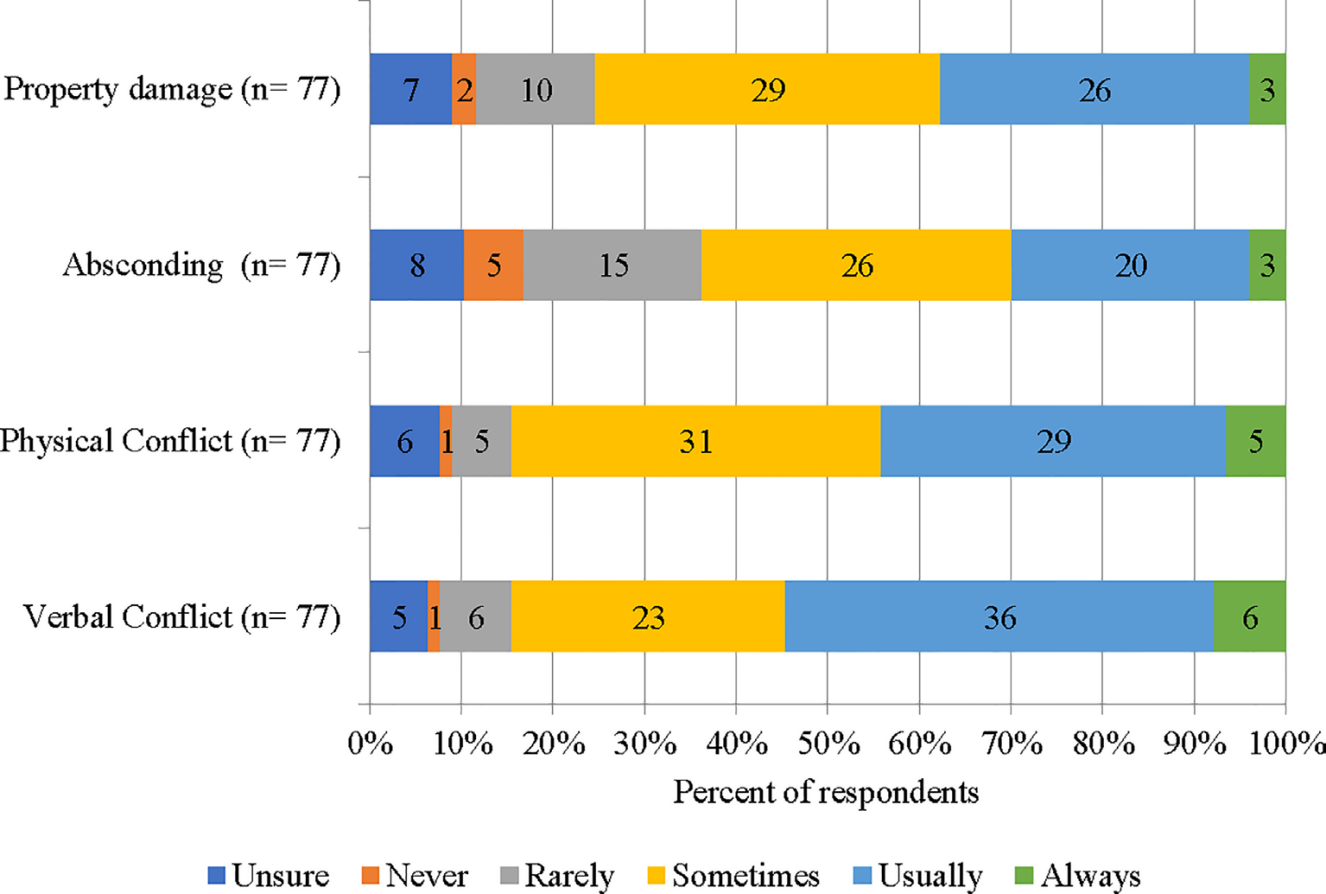
Staff reports of the impact of Safewards on conflict events.

**Figure 1** displays four conflict events and the corresponding rating of staff regarding the impact of Safewards on these. Staff were conservative about the impact of Safewards on absconding and property damage, reporting that Safewards usually or always positively impacted (30% and 35%, respectively). A small group of staff reported that they were unsure or it never had an impact on absconding and property damage. In contrast, staff were clearer that Safewards impacted on physical and verbal conflict, with 45% and 55%, respectively, reporting that Safewards usually or always had a favorable impact.

**Figure 2:**
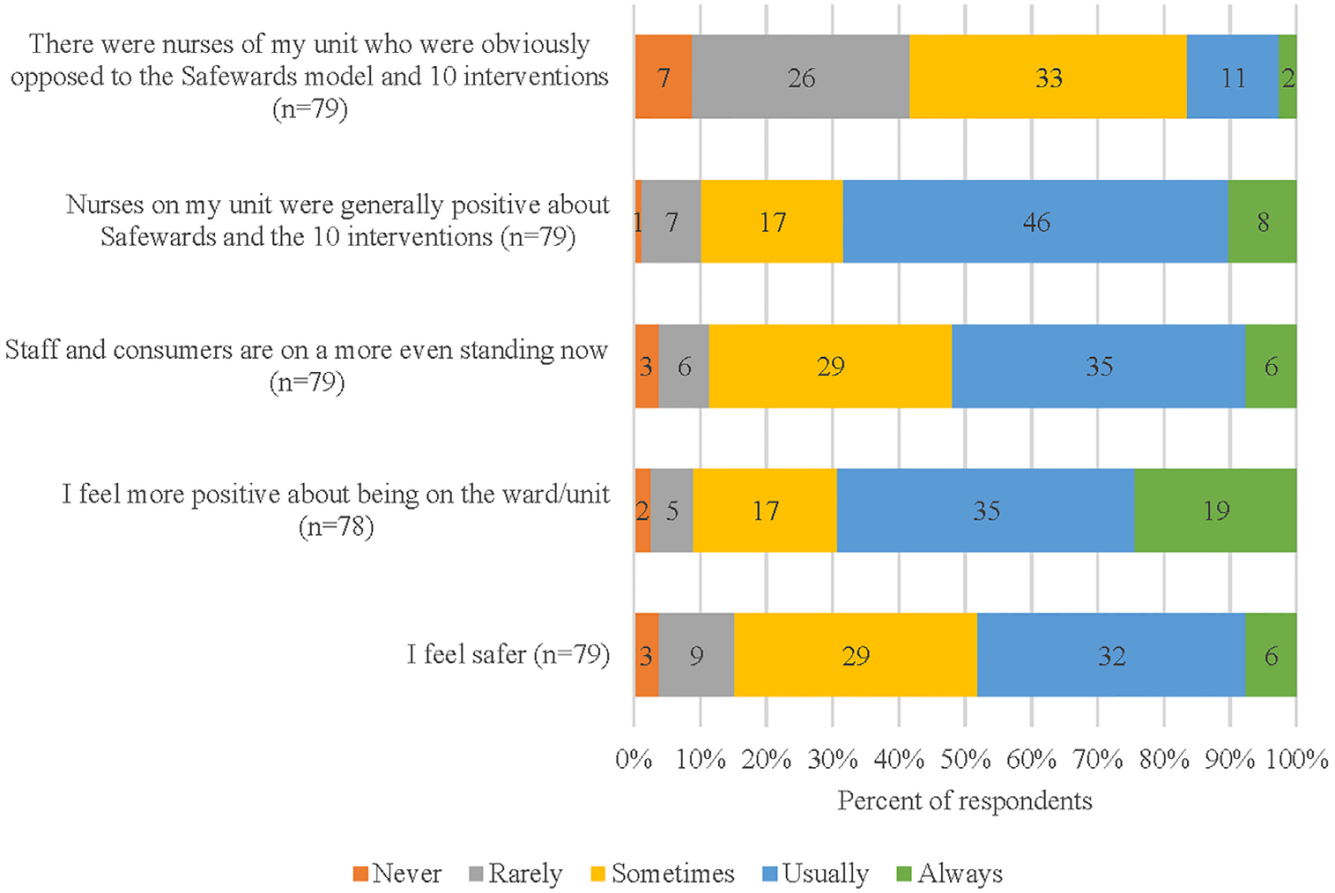
Staff reports of the impact of Safewards on the feel of the ward.

**Figure 2** displays five statements about staff’s experiences of being “on the ward” while Safewards was being implemented. Most staff reported that the nurses were positive about the introduction of Safewards, with a minority reporting that some nurses in their ward were actively opposed to Safewards. Staff felt safer in the ward (50% usually or always) and more positive about being in the ward. Most staff believed that staff and consumers were “on a more even standing” (90% sometimes–always).

### Qualitative Responses

The following provides a thematic analysis of the responses provided by staff. Staff rated interventions as “poor” between 1% and 5% of the time, and six staff provided 13 written comments about their responses. Two themes describe the “poor” rating, *incompatible* and *procedural concerns*. The first theme highlights a staff view that the intervention is *incompatible* with nursing roles and responsibilities. For example, a staff member has a sense that their responsibility is greater than the patient’s, and therefore, the interventions are inappropriate.

The second theme relates to *procedural concerns*, with some respondents reporting that the intervention was poor because there was no ownership taken for the intervention among the team. The critical comments from staff came from a small subset (n = 6) of participants, who were also likely to rate multiple interventions as “poor” or “fair.”

Four key themes summarize the detailed responses of staff regarding their rating of the model or any of the interventions as “excellent.” The themes are *structured and relevant, conflict prevention and reducing restrictive interventions, ward culture change, and promoting recovery principles*. Illustrative quotes are presented in **Table 4**. These four themes incorporate the views of 39 staff with 176 comments related to both the model and all 10 interventions.

**Table 4:** Staff quotes related to themes and specific Safewards interventions or the model.

Theme	Model/Intervention	Quote
Structured and Relevant	Positive Words	“In an inpatient environment where there is a lot of negativity, utilizing positive words (especially during handover and in clinical interactions with other staff) created a more professional, supportive and ‘positive’ workplace.”
	Model	“It feels like we desperately needed something to remind us why we got into this nursing, it brings it back to basics and it brings it back to the patient.”
	Model	“Easy to implement and adopt to current practice, useful for positive patient outcomes.”
	Model	“Easy to follow and helps to keep the ward running smoothly and calmly.”
Conflict prevention and reducing restrictive practices	Model	“Model guides practice and helps us to understand the relationship between conflict and containment.”
	Soft Words	“Sometimes it isn’t what you say that is important rather what you don’t say, staff don’t need to have the last word but need to be there to listen. How you say something considering tone of voice and body language means so much.”
	Talk Down	“This intervention has been helpful in re-educating staff about steps to help reduce/prevent an escalation in client’s behavior so as to reduce the possibility of further restrictive interventions.”
	Talk Down	“Structured process, easily to follow, assisted in reducing restrictive interventions, increased confidence.”
Ward culture change	Model	“It feels like we desperately needed something to remind us why we got into this nursing, it brings it back to basics and it brings it back to the patient.”
	Clear Mutual Expectations	“Collaborative—helped create a sense of community on the unit.”
	Know Each Other	“On this unit we needed something to break down the barriers between patients and staff and it reminds you that you have common ground not only with patients but with each other. It allows us to focus on what we have in common rather than our difference.”
	Know Each Other	“Works well to reduce detachment between patients and staff and to build rapport.”
	Mutual Help Meeting	“This is awesome because it makes it about the patient and empowers them to have a say, and to be a part of what goes on around them during a time when a lot of choice is taken away.”
	Positive Words	“If attitudes start with staff, consumers will reap the rewards making it an easier day for all.”
	Positive Words	“The shift in culture and the shift in language used has been amazing. Staff attitudes have changed dramatically, and for the better.”
Promoting recovery principles	Soft Words	“Utilizes principles of respect and humanity.”
	Positive Words	“This shows respect for our clients and focuses on the positive gains which clients are making in their recovery.”
	Calm Down Methods	“This is very relevant to this unit because we recently had a sensory room put in and we have been using this method with the high dependency patients as well as on the low dependency side. It also has given staff a chance to engage with patients when before we may not have had to try to think of other ways to deescalate.”
	Mutual Help Meeting	“This intervention has helped change the focus from what staff can do for the client, to one where the client is more involved in the decision making about their treatment.”
	Calm Down Methods	“This intervention has been very helpful and is used widely on the unit by most staff to assist clients in dealing with stressors and gives them some ownership of how they deal and treat issues relating to their situation.”
	Reassurance	“A constructive intervention that offers respect and dignity.”
	Discharge Messages	“Provides messages of hope for other patients, we can direct patients to the tree to show that discharge will happen for them and to hold onto hope.”
	Discharge Messages	“The patients love it. For the patients to be able to read from other patient’s messages of hope is far more powerful than anything that we as nurses can attempt.”

In relation to the theme *structured and relevant*, staff put forward the idea that the model and interventions reminded them of their professional training and refreshed their thinking about providing more holistic care. Specifically, staff reported prioritizing the staff–consumer relationship with Safewards in ways that other ward system models may not. Staff affirmed that the model was clear and simple to follow.

The theme *conflict prevention and reducing restrictive practices* highlights staff’s feedback regarding a renewed understanding of the relationship between conflict and containment, resulting in increased confidence to listen well and talk respectfully to consumers in a way that minimizes frustration and, by extension, interrupts the cycle of conflict and containment.

The staff who highlighted a *positive ward culture change* described less social distance and enhanced mutual regard, arising from sharing responsibility and increased collaboration between staff and patients. A number of mechanisms related to specific interventions facilitated this culture change, for example, Know Each Other helped staff and consumers to find commonality with each other, Clear Mutual Expectations increased the sense of community in the ward, Positive Words led to attitude changes in staff, which in turn improved their interactions with consumers.

The theme *promotes recovery principles* captures the feedback from staff that a variety of interventions enhances consumer involvement in their care and treatment, hope and peer support, choice, dignity, and respect from staff toward consumers.

## Discussion

This paper reports on staff experiences and views of the suitability and impact of the Safewards theoretical model and its 10 interventions. Overall, staff reported that Safewards impacted on physical and verbal conflict and supported a positive change in the ward environment and relationships, helping staff to feel safer. The following sections discuss the current findings in relation to whether Safewards reduced conflict events or flashpoints. These findings can be contrasted with those obtained from consumers (38).

### Reducing Restrictive Practices

Generally, staff indicated that Safewards had impacted on flashpoints in a positive way. Like consumers, the staff were most modest in their views about absconding and property damage. Staff were most confident in the impact of Safewards on verbal and physical conflict, although the staff group had a much stronger view of the impact of Safewards on physical and verbal conflict (45% and 55%, respectively, compared with 25% of consumers usually or always had an impact) (38).

The theme *conflict prevention and reducing restrictive practices* highlights the relational aspects of Safewards. The findings of this study indicate that, in challenging situations, staff feel more empowered and permitted to act with a renewed understanding of the impact of their responses on consumers. This may suggest a positive shift away from the use of restrictive practices to maintain compliance, thus giving consumers the potential to trust the staff more, consequently building relationships (12).

### Shifting Culture and Improving Recovery-Oriented Practice

Staff reported that Safewards had a positive impact on their experience of being in the ward. Both staff and consumers described a more equal relationship as a result. These findings indicate some differences in perceived changes. Consumers were most positive about feeling safer in the wards (95% sometimes or usually); staff were slightly less so (85% sometimes to usually). In contrast, staff were more optimistic than consumers about the shift to a more equal staff–consumer relationship (90% compared to 70% sometimes to always) (38).

The qualitative findings highlighted themes unique to the staff perspective, such as *structured and relevant*. Staff were positive about Safewards legitimizing and operationalizing the centrality of person-centered care. This finding supports previous research that nurses experience increased satisfaction when they are able to spend more time in direct interactions with their patients (20). These opportunities need to be built into ward routines. Safewards was viewed by most as feasible in the current practice environment of competing demands (25). Positive words specifically helped create a professional, supportive, and positive workplace. This finding may highlight one of the key drivers in our previous findings regarding a reduction in seclusion rates associated with Safewards. Previous research has found that negative staff morale increases the likelihood of conflict and containment; these were decreased when staff engaged in positive practice, such as being compassionate and valuing consumers (40).

Staff in this study highlighted that Safewards is clear and straightforward to understand and implement. This finding is at odds with other studies that have found staff did not readily accept or adhere to the interventions and, consequently, fidelity was poor (33, 34). In contrast, in Victoria, before implementation, staff in all wards participated in Safewards training, with evaluation surveys revealing significant increases in staff knowledge, confidence, and motivation to implement Safewards (Fletcher et al., submitted). This provides a possible explanation about staff understanding of the Safewards model and interventions and may explain the high fidelity scores achieved. Furthermore, staff in the present study reported that it was highly probable Safewards would be sustained in their health services over the next 12-months (2 years after Safewards was first implemented). Together, these findings provide support for the notion that Safewards has the potential to be sustained long-term and highlights some factors that may be key to achieving this sustainability (37).

*Ward culture change* relates to changes in staff attitudes toward consumers and building rapport, which stems from staff realizing and accepting that they are in a position to influence most aspects of the ward procedures and interactions. This corresponds to conclusions drawn in previous research that views about restrictive practices are divergent among staff. When staff connect with the uniqueness of a consumer, they are less likely to believe in the use of restrictive practices. In contrast, when distance remains between staff and consumers, staff view consumers as having “common needs and common restrictions” (8).

Feedback from staff in the current study aligns with consumer feedback regarding Safewards promoting aspects of recovery. In particular, consumers consulted in our work have expressed a view that Safewards promotes respect for consumers, enhancing consumer participation in their care, and the importance of dignity and hope (38).

### Perceived Shortcomings

A small minority of staff rated the model or interventions as poor. Reasons provided included describing a lack of staff ownership resulting in the intervention not being implemented well. This theme aligns with the small group of consumers who rated some of the interventions poorly, with one reason being that staff did not use the interventions in some wards (38). Furthermore, staff rated some of the interventions as poor either because they believed that staff had more responsibility than consumers and therefore an intervention that attempted to level this out was viewed as problematic or because consumers were too unwell to use the intervention appropriately thus it was incompatible. These were not concerns experienced by the consumers (38).

### Limitations

The current study may have included a biased sample as staff self-selected, so those with more positive views may have been more inclined to participate. Although all services were represented, the distribution was not representative of the number of wards involved in each service.

## Conclusion

The present study suggests that the feasible and simple implementation of Safewards has had a positive and pervasive impact on the experience of staff in acute wards across Victoria. Quantitative data showed that staff identified the Safewards model and interventions as having a role in reducing physical and verbal conflict in wards and resulted in staff feeling safer. Qualitative data highlighted that staff experienced a shift in culture, resulting in better relationships with consumers and between staff, as well as a renewed focus on patient-centered, recovery-oriented care. Staff in particular described a less uneven relationship with consumers, suggesting that Safewards has an impact on power dynamics that has previously been linked to the use of restrictive interventions (8). Previous research has highlighted that, when staff are custodial rather than caring, the rate of incidents is higher and so is the potential for use of containing or restrictive interventions (41). A significant investment has been made in Australia in attempting to reduce restrictive interventions over the past two decades through law, policy, and practice change. Safewards appears to support these efforts and needs to be consistently implemented with fidelity to the model to continue the downward trajectory now observed in publicly available reports (42, 43). By easily fitting into the ward flow, Safewards can provide the increased motivation, momentum, and support for staff to engage with consumers more therapeutically and from a recovery-oriented perspective. Future research should focus on the intersection of Safewards and recovery-oriented practice on staff well-being and experiences at work. Further work is required to understand how Safewards interacts with other ward activities, such as sensory modulation (44, 45) and legislative coercion (46).

## Ethics Statement

This study was conducted in accordance with and after recommendations from Victorian Human Research Ethics Multi-site process (ID 15225L). Participants were provided a Plain Language Statement. Consent was indicated on the first page of the online survey where participants were asked to “Please tick on the following statement to indicate that you have read and understood the participant information.” Completion of online surveys was anonymous. The protocol was approved by the Monash Health Human Research Ethics Committee.

## Author Contributions

JF and BH were involved in the development of the study, data collection, and analysis. JF, BH, and LB were involved in the interpretation of data. JF, BH, SK, and LB were involved in the writing and editing of the manuscript.

## Funding

This paper forms part of the work toward a PhD, which is supported through an Australian Government Research Training Program Scholarship. JF is supported by NHMRC PhD Research Scholarship 1133627. SK is supported by NHMRC Research Fellowship APP1078168. The Department of Health and Human Services, Government of Victoria funds clinical services across the state. This independent evaluation was financially supported by the Office of the Chief Mental Health Nurse, in the Department of Health and Human Services, Government of Victoria.

## Conflict of Interest Statement

The authors declare that the research was conducted in the absence of any commercial or financial relationships that could be construed as a potential conflict of interest.
